# A novel approach to assess surface roughness and EDX profiling of blue rotary NiTi files following dynamic immersion in various hypochlorite concentrations

**DOI:** 10.1186/s12903-025-07075-y

**Published:** 2025-11-06

**Authors:** Hebatullah Ahmad Safwat, Nesreen Y. Mohammed, Asmaa Abd El-Hady

**Affiliations:** 1 Department of Endodontics, Faculty of Dental Medicine for Girls, Al-Azhar University, Cairo, Egypt; 2 Department of Dental Biomaterials, Faculty of Dental Medicine for Girls, Al-Azhar University, Cairo, Egypt

**Keywords:** Blue heat-treated NiTi files, Surface roughness, Scanning electron microscopy (SEM), ImageJ software, NaOCl

## Abstract

**Background:**

Successful root canal treatment relies on effective chemomechanical preparation. However, sodium hypochlorite (NaOCl), the standard irrigant, can corrode metals and weaken the structural integrity of nickel-titanium (NiTi) instruments. Therefore, this study aimed to evaluate the surface roughness and element composition of Fanta AF blue and E3 Azure NiTi rotary endodontic files after dynamic immersion in two different NaOCl concentrations.

**Methods:**

Forty-two heat-treated rotary files were divided into two groups based on the file brand, Fanta AF Blue and E3 Azure NiTi files. Each group was randomly subdivided into three subgroups (n = 7) according to irrigant exposure: Control (no exposure), 5.25% and 2.6% NaOCl. The surface morphology and elemental composition of new files and those immersed in NaOCl for 10 min were analyzed using high-resolution scanning electron microscopy (SEM) and energy-dispersive X-ray microanalysis. SEM images were analyzed with Image J 1.52 software to determine arithmetic mean roughness (Ra) and root mean square roughness (Rq). When the ANOVA test showed significance (p ≤ 0.05), pairwise comparisons were made using Bonferroni’s post-hoc test.

**Results:**

Fanta files immersed in 2.6% NaOCl exhibited significantly higher mean Ra and Rq values than Azure files (*p* = 0.020 and 0.022, respectively). Immersing Fanta files in 2.6% NaOCl resulted in a significantly greater weight percentage (wt.%) loss of Titanium (*p* = 0.009) and Nickel (*p* = 0.021) compared to new Azure files and those immersed in 5.25% NaOCl. The carbon wt.% significantly increased in Fanta files immersed in 2.6% NaOCl (*p* = 0.014).

**Conclusions:**

Within the limitations, Azure files showed lower overall surface roughness than Fanta files after dynamic immersion in 2.6% NaOCl. The undesired effects of 2.6% NaOCl on Fanta files could lead to crack propagation and decreased cyclic fatigue resistance, which requires further research.

**Graphical abstract:**

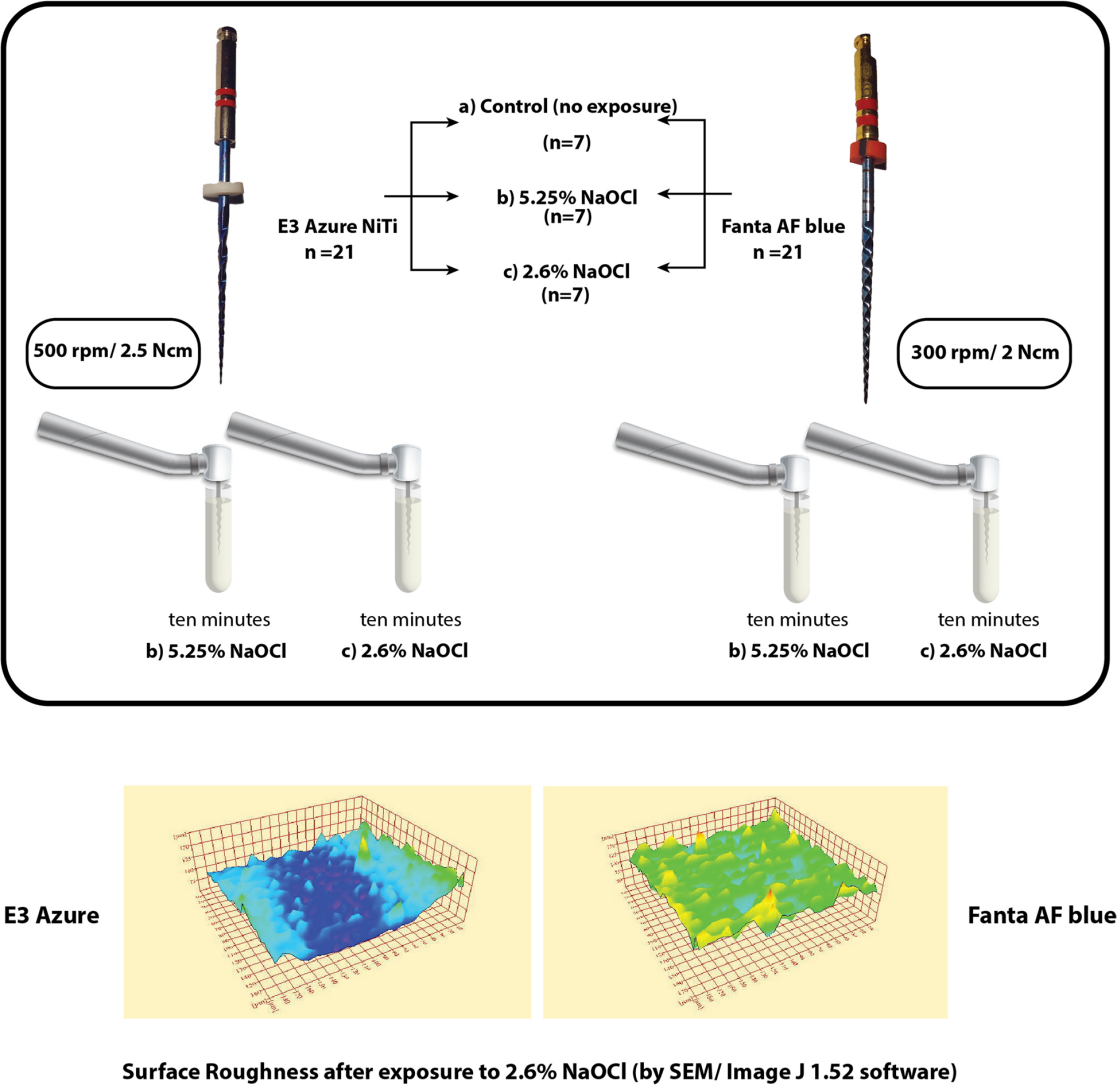

## Background

Chemomechanical preparation is fundamental for effective root canal therapy. It involves using endodontic files to clean and shape the root canal system, along with irrigants for disinfection. Sodium hypochlorite (NaOCl) is the preferred irrigant because of its ability to dissolve soft tissue and its powerful, broad-spectrum antibacterial properties. However, NaOCl is a strong oxidizing agent with high alkalinity that can corrode metals, potentially causing significant damage to the mechanical and structural qualities of endodontic files [1–3].

Nickel-titanium (NiTi) files have evolved significantly over the past two decades, with notable changes in shape and metallurgical features since their introduction by Walia et al. in 1988. However, several innovative thermomechanical treatments have been applied to conventional NiTi alloy files to improve their mechanical properties and modify their phase transition behaviour [4, 5]. Fanta AF blue rotary file (Shanghai Fanta Dental Materials Co., Ltd.) is made of specially heat-treated AF wire (AFTM-H) [6], whereas Endostar E3 Azure file (Poldent, Warsaw, Poland) has undergone a heat treatment procedure given by a highly advanced technology known as "Azure HT technology" [7]. This treatment forms a titanium oxide layer that gives these files their characteristic blue color, similar to vortex blue, enhancing both cutting efficiency and wear resistance [8].

The surface roughness of NiTi endodontic files is a crucial property that influences the fracture mechanism of these files. Studies assessing the surface roughness have employed non-contact three-dimensional optical profilometry, atomic force microscopy, and a combination of scanning electron microscopy (FE-SEM) with ImageJ software [9–12]. This analysis is based on SEM evaluation because it allows for detailed structural analysis through secondary electron imaging and can detect most endodontic file flaws at high resolution [13]. As more NiTi instruments with newly developed proprietary heat treatment technologies enter the market and are commercialized worldwide, further research is needed to understand better their mechanical properties, as well as the elemental and morphological changes that can occur when in contact with common endodontic irrigants, such as NaOCl.

Therefore, the purpose of this study was to evaluate the surface roughness and element composition of AF Blue and E3 Azure NiTi rotary endodontic files after they underwent dynamic immersion in two different concentrations of NaOCl. This analysis was conducted using scanning electron microscopy and Image J. The null hypothesis stated that there would be no significant differences in surface roughness or element composition between AF Blue and E3 Azure files before and after immersion in 5.25% and 2.6% NaOCl solutions.

## Methods

This study employed commercially available heat-treated endodontic NiTi files and received ethical approval from the Research Ethics Committee of the Faculty of Dental Medicine for Girls et al.-Azhar University, Cairo, Egypt (REC-PD-25–03).

### Sample size calculation

The sample size was determined using Cai et al. [14] and the G power statistical power analysis tool (version 3.1.9.4). To detect a large effect size (f) = 0.9 with an actual power (1-β error) of 0.8 (80%) and a significance level (α error) of 0.05 (5%) for a two-sided hypothesis test. A total sample size of 42 files, with 21 in each group based on the brand, divided into three subgroups (n = 7) for control and two different irrigation concentrations, was deemed sufficient.

### Sample grouping

A total of forty-two heat-treated endodontic rotary NiTi files were divided into two groups (*n* = 21) based on the file brand: Fanta AF Blue and E3 Azure, both with a 25/0.06 taper. Each group was further randomly divided into three subgroups (n = 7) based on the type of irrigant exposure: control (no exposure), 5.25% NaOCl concentration, and 2.6% NaOCl concentration.

### Dynamic immersion procedure

**Fig. 1:**
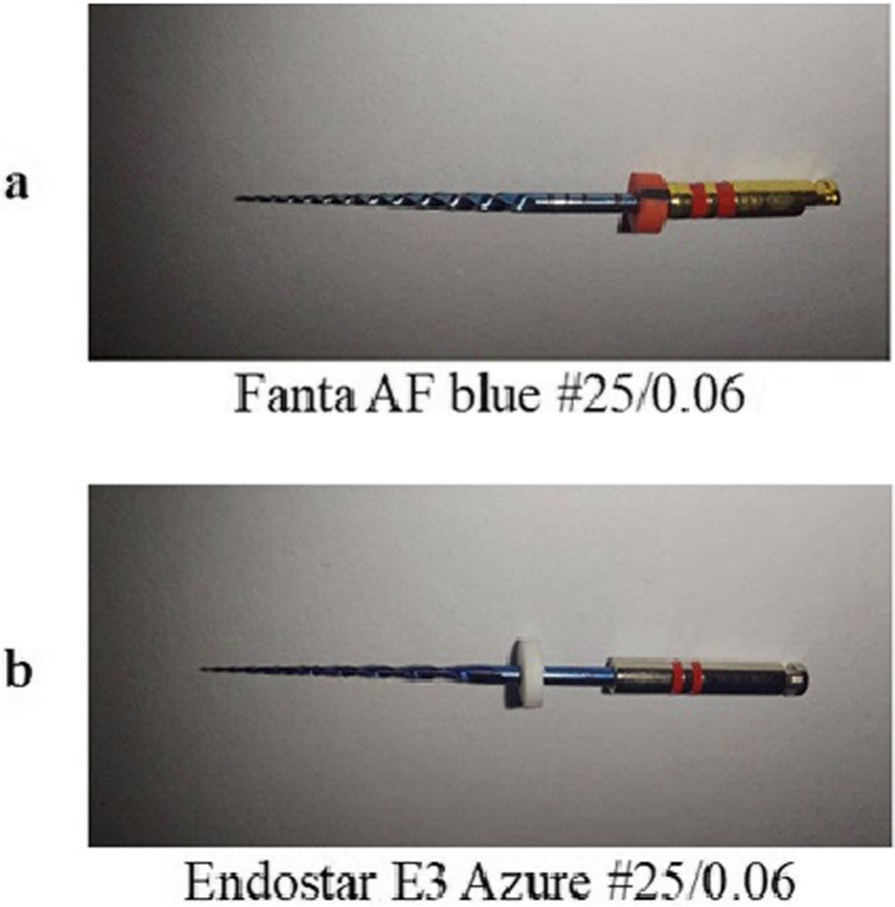
Photographs of the AF blue NiTi rotary endodontic file (**a**) and the E3 Azure NiTi rotary endodontic file (**b**) used in the study

An endodontic motor (Marathon E class, Saeyang, South Korea) was used to rotate the Fanta AF blue (Lot: 112,211,110,005) and Endostar E3 Azure files (Lot 173,919) as shown in Fig. 1. Each file in the experimental groups was individually immersed in two NaOCl (Clorox—Household Cleaning Products of Egypt) solutions with concentrations of 5.2% and 2.6% at room temperature. A 2.6% concentration was prepared by diluting 5.25% NaOCl at a 1:1 ratio using distilled water. AF Fanta Blue and E3 Azure files were dynamically immersed using an endodontic motor operated at their specified speeds and torques: 300 rpm/2 Ncm and 500 rpm/2.5 Ncm, respectively. The immersion lasted ten minutes, with the NaOCl solution replaced at each file change. Afterward, the file was rotated in distilled water for ten minutes. To prevent galvanic corrosion, the irrigating solution was not allowed to come into contact with the file handle [15, 16].

### Scanning electron microscopy (SEM) evaluation and imageJ analysis

The surface morphology and elemental composition of each new sample and those immersed in NaOCl were examined using high-resolution scanning electron microscopy (SEM) imaging and energy-dispersive X-ray microanalysis (EDX) with SEM (Quanta FEG 250, FEI, USA). Two authors, one specialized in endodontics and the other in dental biomaterials, independently selected sites from the observed defective areas on the SEM image while blinded to file brand and irrigant exposure. In cases of disagreement, they discussed the images until a consensus was reached.

Images were taken at two magnifications: 100 × and 400x, approximately 4 mm away from the file tip. They were captured in low vacuum/ESM modes with an excitation voltage of 20.00 kV and a working distance of 12–18 mm, using a large-field detector. ImageJ 1.52 (U. S. National Institutes of Health, Bethesda, MD, USA) was used to analyze SEM images and calculate metrics such as arithmetical mean roughness (Ra) and root mean square roughness (Rq) [13]. For each scanned image, parameter values were obtained from two sites (159.8 µm × 172.7 µm) and averaged.

### Statistical analysis

The statistical analysis was performed using IBM SPSS Statistics for Windows, Version 23.0. Armonk, NY: IBM Corp. The distribution of numerical data was tested for normality using Kolmogorov–Smirnov and Shapiro–Wilk tests. All data followed a normal distribution and were presented as mean and standard deviation (SD) values, then analyzed using multivariate ANOVA. Bonferroni's post-hoc test was used for pairwise comparisons when the ANOVA was significant. The significance level was set at p ≤ 0.05.

## Results

**Fig. 2:**
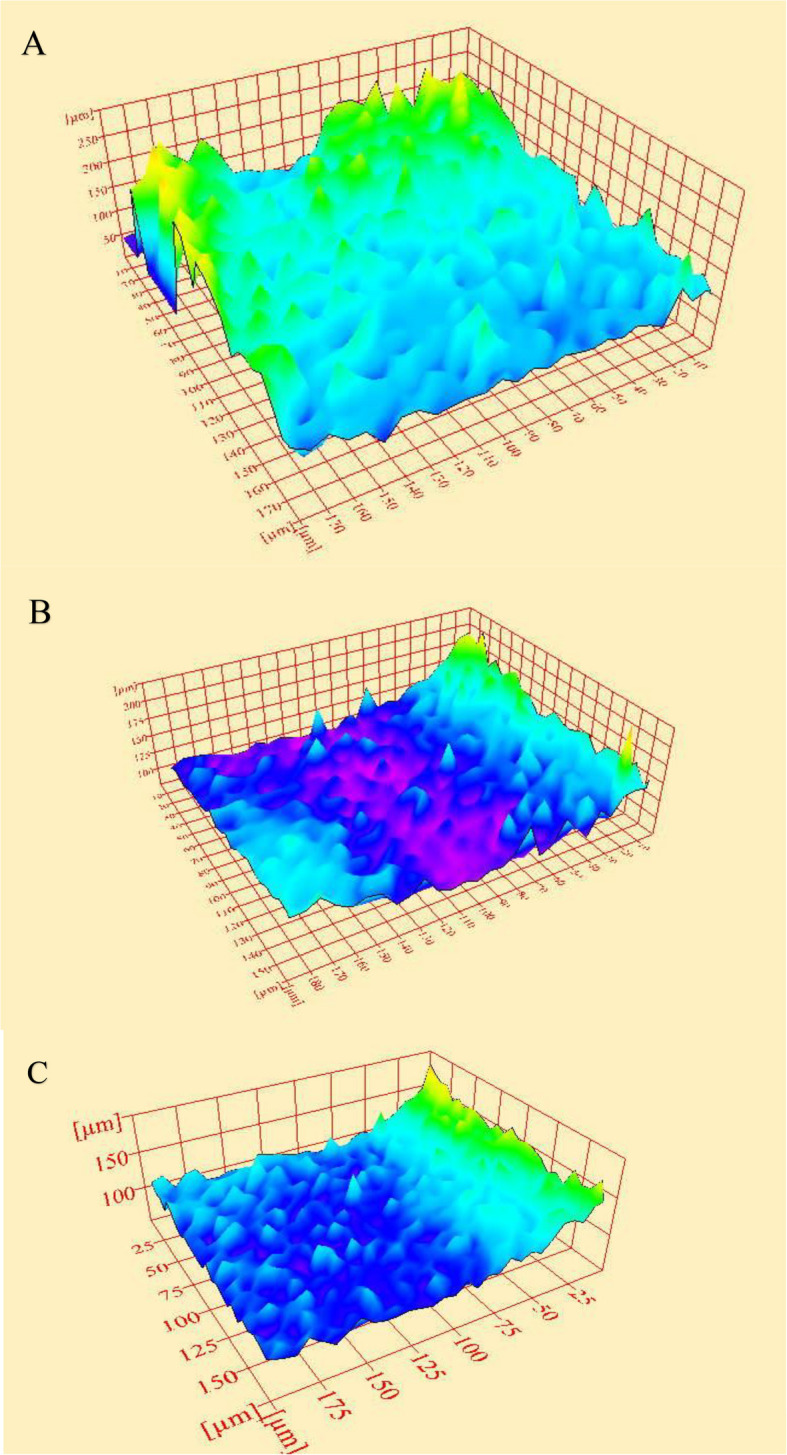
3D images of the surface morphology of E3 Azure NiTi rotary endodontic files. **A** Before dynamic immersion in various hypochlorite concentrations. **B** After 10 min of dynamic immersion in NaOCl at 5.25% concentration. **C** After 10 min of dynamic immersion in NaOCl at 2.6% concentration

**Fig. 3:**
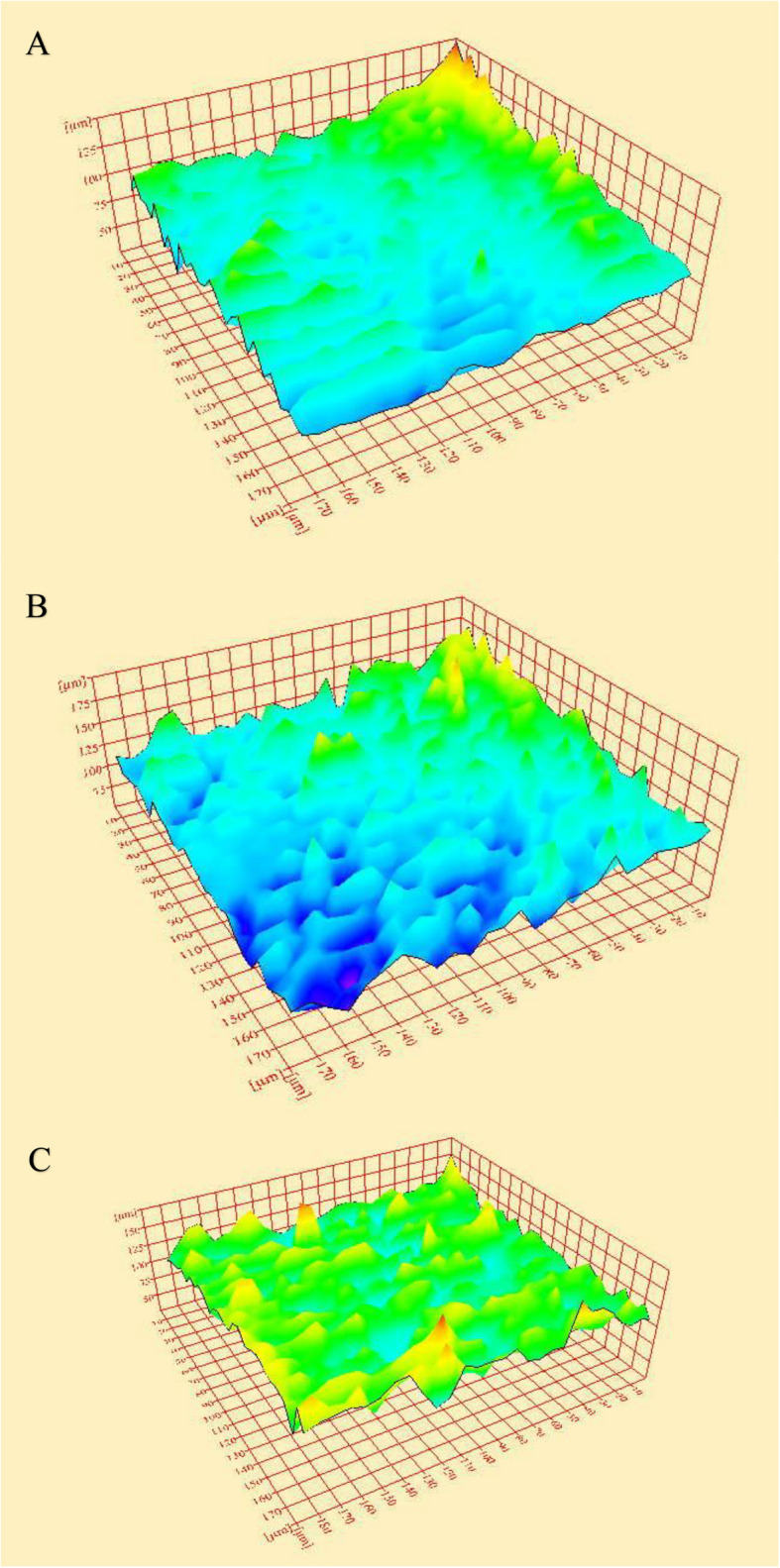
3D images of the surface morphology of AF blue NiTi rotary endodontic files. **A** Before dynamic immersion in various hypochlorite concentrations. **B** After 10 min of dynamic immersion in NaOCl at 5.25% concentration. **C** After 10 min of dynamic immersion in NaOCl at 2.6% concentration

The mean and standard deviation values for Ra and Rq surface roughness parameters of AF blue and E3 Azure NiTi rotary endodontic files following dynamic immersion in various NaOCl concentrations are shown in Table 1. There was a significant difference between the two file brands after immersion in 2.6% NaOCl in surface roughness values. Compared to Azure files, Fanta files showed a significant increase in mean Ra and Rq values after immersion in 2.6% NaOCl (p = 0.020 and 0.022, respectively). Although Fanta files also demonstrated increased mean Ra and Rq values following immersion in 5.25% NaOCl rather than Azure, the difference was not statistically significant. The representative 3D images of the surface morphology of both E3 Azure and AF blue files for control and those dynamically immersed in different NaOCl concentrations are displayed in Figs. 2 and 3, respectively.

**Table 1:** Comparison between Ra and Rq (µm) roughness parameters with different interactions of variables

Measurement	Irrigant concentration	Azure		Fanta		*P*-value	Effect size *(Partial eta squared)*	95% CI for effect size
		Mean	SD	Mean	SD			
Ra	New Files	9.08	0.97	9.02	1.53	0.938	0.0002	(0 – 0.045)
	5.25%	9.39	1.33	9.45	2.09	0.945	0.001	(0 – 0.041)
	2.6%	8.13	0.64	10.2	2.28	0.020^*^	0.142	(0.002 – 0.344)
	*p*-value	0.313		0.380				
	Effect size *(Partial eta squared)*	0.062		0.052				
	95% CI for effect size	(0 – 0.219)		(0 – 0.203)				
Rq	New Files	12.69	1.36	12.19	2.17	0.644	0.006	(0 – 0.131)
	5.25%	12.85	1.59	12.98	2.68	0.901	0.0004	(0 – 0.068)
	2.6%	11.30	0.98	13.86	2.65	0.022^*^	0.137	(0.001 – 0.338)
	*p*-value	0.295		0.308				
	Effect size *(Partial eta squared)*	0.066		0.063				
	95% CI for effect size	(0 – 0.224)		(0 – 0.221)				

Descriptive statistics and multivariate ANOVA results comparing means and standard deviations before and after dynamic immersion in NaOCl for ten minutes

^*^Significant at *p* ≤ 0.05

Furthermore, immersing Fanta files in NaOCl at a 2.6% concentration resulted in a significantly higher weight percentage (wt.%) loss of Titanium and Nickel elements compared to new Fanta files and those immersed in 5.25% concentration (p = 0.009 and 0.0210, respectively; Table 2).

**Table 2:** Surface elements wt.% of Azure and Fanta files with different interactions of variables

Element	Irrigant concentration	Azure		Fanta		*p*-value	Effect size *(Partial eta squared)*
		Mean	SD	Mean	SD		
Carbon	New Files	1.82	0.48	2.13^B^	0.57	0.293	0.031
	5.25%	2.21	0.62	2.11^B^	0.37	0.752	0.003
	2.6%	2.33	0.41	2.91^A^	0.74	0.057	0.097
	*p*-value	0.197		0.014^*^			
	Effect size *(Partial eta squared)*	0.086		0.211			
Oxygen	New Files	6.75	1.12	5.1^B^	1.62	0.026^*^	0.131
	5.25%	4.98	1	7.69^A^	1.46	< 0.001^*^	0.29
	2.6%	5.64	1.06	5.52^B^	1.54	0.864	0.001
	*p*-value	0.053		0.002^*^			
	Effect size *(Partial eta squared)*	0.151		0.3			
Titanium	New Files	39.73	0.71	38.14 ^A^	2.18	0.361	0.023
	5.25%	37.08	2.4	38.99 ^A^	0.88	0.274	0.033
	2.6%	35.77	4.59	33.71 ^B^	5.38	0.237	0.039
	*p*-value	0.077		0.009^*^			
	Effect size *(Partial eta squared)*	0.133		0.233			
Nickel	New Files	48.77	1.11	46.86 ^A^	2.87	0.424	0.018
	5.25%	45.25	3.58	47.79 ^A^	1.02	0.290	0.031
	2.6%	43.23	6	41.34 ^B^	7.65	0.430	0.017
	*p*-value	0.074		0.021^*^0			
	Effect size *(Partial eta squared)*	0.135		0.194			

Descriptive statistics and results of Multivariate ANOVA test for comparing the weights of Carbon, Oxygen, Titanium, and Nickel %

^*^Significant at *p* ≤ 0.05, Different superscripts in the same column indicate statistically significant difference between NaOCl concentrations

**Fig. 4:**
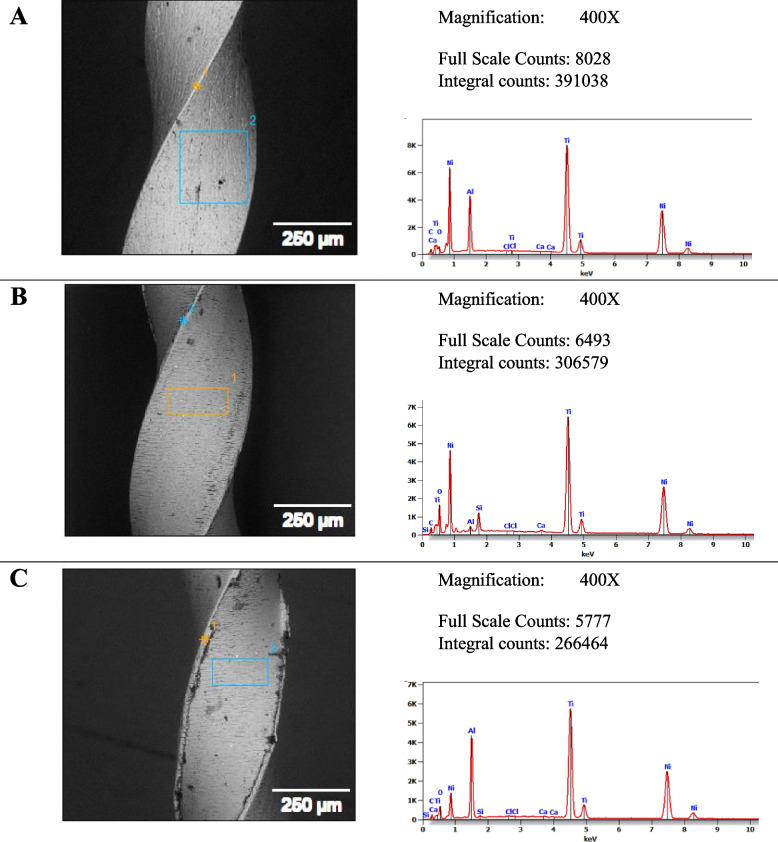
Representative SEM micrographs (400X) showing surface morphology with the EDX spectra and elemental mapping for selected areas of the Fanta files showing mainly Ni and Ti, with minor amounts of O and C. **A** Control (no exposure), **B** After 10 min dynamic immersion in 5.25% hypochlorite concentration, and **C** After 10 min dynamic immersion in 2.6% hypochlorite concentration. EDX microanalysis is conducted in conjunction with the Quanta FEG 250 SEM, ensuring consistency in parameters and conditions across images. Variations in contrast and brightness settings, and scale within the software, may describe differences between SEM images accompanied by EDX analysis and those captured originally by SEM

**Fig. 5:**
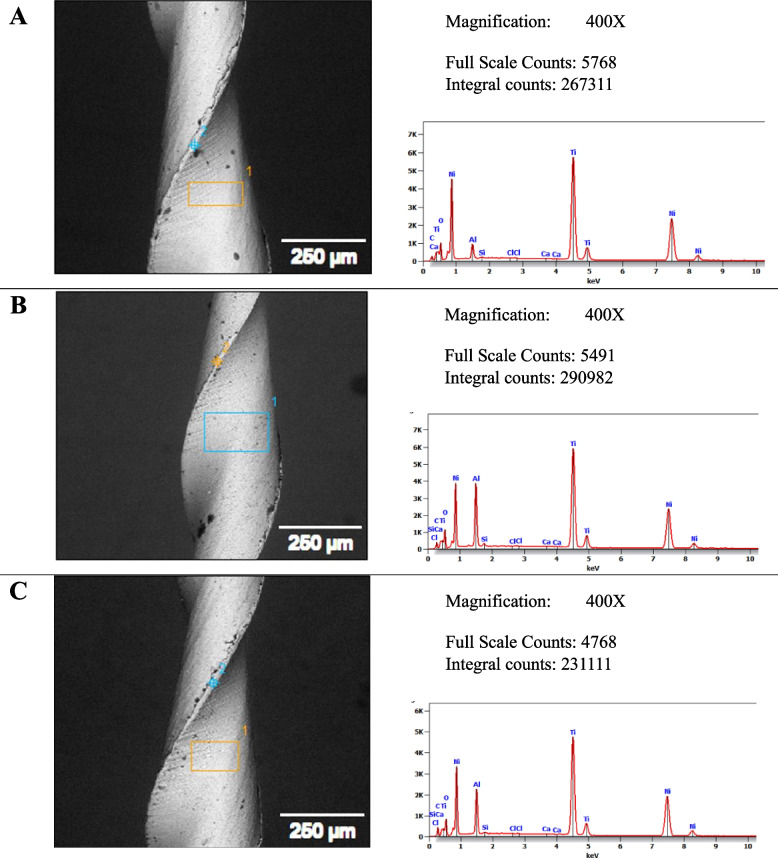
Representative SEM micrographs (400X) showing surface morphology with the EDX spectra and elemental mapping for selected areas of the Azure files showing presence mainly Ni and Ti, with minor amounts of O and C.** A** Control (no exposure), **B** After 10 min dynamic immersion in 5.25% hypochlorite concentration, and **C** After 10 min dynamic immersion in 2.6% hypochlorite concentration. EDX microanalysis is conducted in conjunction with the Quanta FEG 250 SEM, ensuring consistency in parameters and conditions across images. Similar to Fig. 4, the EDX analysis aligns with the SEM images, and differences in software settings may account for the discrepancies between the images accompanied by EDX analysis and those captured originally by SEM

Results of representative SEM micrographs with EDX spectra of the Fanta files following exposure to the irrigant are shown in Fig. 4. While those of Azure files are displayed in Fig. 5. Elemental composition analysis showed that Carbon wt.% increased significantly in the Fanta files group after immersion in a 2.6% concentration (*p* = 0.014; Table 2). Additionally, Azure files lost more Titanium and Nickel after immersion in a 2.6% NaOCl solution compared to new Azure files and those immersed in a 5.25% concentration. However, the difference was not statistically significant.

## Discussion

NiTi endodontic instruments are susceptible to corrosion during chemomechanical preparation, chemical disinfection, or sterilization. Many studies have shown a correlation between reduced cutting efficiency, increased surface microcracks, micro-pitting, and corrosion areas [14, 17–19]. Corrosion patterns that selectively remove nickel from the surface, resulting in micro-pitting, can induce stress accumulation and crack formation, weakening the instrument's structure [20–22]. Pitting or crevice corrosion might occur first and lead to fatigue failure, shifting the fracture mode from traditional fatigue to corrosion failure [23].

Sodium hypochlorite (NaOCl), the gold-standard irrigating solution, is a chlorine-containing solution that is corrosive to metals and may weaken the structural integrity of a NiTi instrument during instrumentation or disinfection [1]. Therefore, this study aimed to evaluate the surface roughness and element composition of AF Fanta blue and Azure NiTi rotary endodontic files following dynamic immersion in NaOCl at 5.25% and 2.6% concentrations. The null hypothesis was partially rejected.

Dynamic immersion was carried out in a plastic container to simulate the dynamics of clinical practice, eliminate instrument friction with root canal models, and ensure consistent experimental comparisons. A 5.25% NaOCl solution was used because it is commonly applied in clinical settings. However, testing the instruments at lower concentrations might improve their surface properties and overall performance. Additionally, it has an effective antimicrobial action that eliminates bacteria in the root canal system while showing reduced cytotoxicity. Therefore, it helps to lower the risk of tissue irritation or damage [2, 3]. Therefore, it helps to lower the risk of tissue irritation or damage. Consequently, 5.25% NaOCl was diluted one-to-one, resulting in a 2.6% NaOCl concentration [24]. Although files are not continuously rotated in the canal for 10 min, such an immersion protocol appears suitable in the current study. It aligns with clinical conditions during root canal treatment, where preparation times typically range from 12 to 40 min. These times may vary depending on the instruments employed and specific anatomical features of the root canals, including curvature, length, and width [25]. Additionally, a recent study utilised the same file to prepare three canals, each for two minutes [26].

Endodontic instrument surface roughness can be assessed using various methods and parameters, with Ra and Rq being the most frequently used [27]. Previous research has confirmed the accuracy and reliability of using ImageJ software to reconstruct 3D images from FE-SEM data and measure roughness parameters of the scanned surface [13, 15, 28]. In the present study, the reconstruction of 3D images and the measurement of Ra and Rq surface roughness parameters were performed using ImageJ 1.52 software. They were based on data acquired from images obtained with the Quanta FEG 250-SEM. The selection of defective areas on the SEM images was performed by two examiners blinded to file brand and irrigant exposure. Although blinding was employed to minimize potential bias, the process inevitably relied on the subjective judgment of the examiners. This subjectivity represents a methodological limitation that should be considered when interpreting the findings.

New files from both brands showed surface flaws, with Azure exhibiting higher Ra and Rq values than Af Fanta blue; however, the difference was not statistically significant. This might be due to the manufacturing process. These findings align with previous research, which reported that machining of rotary NiTi instruments often produces an irregular, stressed surface. Additionally, plastic deformation, characterized by milling grooves, pits, fissures, and regions of metal rollover, was produced [19]. Both files exhibited an increase in topographical irregularities at the microscale level after dynamic immersion in NaOCl irrigant at concentrations of 5.25% and 2.6%, due to the corrosive effect of the irrigant.

The performance of endodontic instruments in NaOCl solutions can differ because of factors such as alloy properties, instrument design, and rotational speed [24]. After ten minutes of dynamic immersion in NaOCl at 2.6% concentration, the mean Ra and Rq values of Fanta files increased significantly. These unexpected results can be attributed to the metallurgical properties of the newly developed heat-treated alloy, AF-H wire, from which the Fanta files are fabricated. However, there is currently no published research on this heat treatment. Furthermore, a 2.6% NaOCl concentration might have lower viscosity than 5.25%, potentially enhancing its contact with the specific instrument design and making it more detrimental than the higher concentration. The rotational speed used for the Fanta files may influence the solution temperature surrounding the instrument, which could, in turn, affect its interaction with the file surface [26]. Additionally, differences in alkalinity between irrigant concentrations, along with the undisclosed manufacturing process of Azure files, may hinder surface alterations when the same irrigant is used.

Previous investigations revealed that immersion in NaOCl and EDTA increased the surface roughness of endodontic files because of corrosion [1, 29, 30]. However, directly comparing the results across these investigations is challenging due to differences in the files' manufacturing process, metallurgical structure, surface treatments, pH levels of the testing solutions, and evaluation methods.

NiTi alloys were known to contain approximately 55% Ni and 45% Ti, which accounts for the alloy's superior elastic characteristics [31]. Titanium is an allotropic metal having compact hexagonal (α, austenite) or body-centered cubic (β, martensite) structures. Titanium alloying elements are either neutral (α-phase stabilisers) or alphagenic (β-phase stabilisers) based on the stabilising effects of their respective phases. Aluminium (Al) is the most common alphagenic alloying element; however, carbon (C), oxygen (O), and nitrogen (N) can also be used [32]. EDX microanalysis is recognized as a credible method to assess the chemical composition of the file alloy [33]. In this study, EDX analysis verified that both brands of files were primarily composed of Ni and Ti, with minor amounts of O and C, resulting in a more martensitic crystalline structure. However, the low wt.% of O and C in new files from both brands suggests that these elements are present in modified oxide layers rather than as components of the manufactured alloy itself. The Ti oxide layer on the surface of both brands of files gives them a characteristic blue colour and was claimed to contribute to their corrosion resistance. At the same time, the presence of C may suggest a surface contaminant acquired during the manufacturing process. New Azure files exhibited a significantly higher mean O wt.% than new Fanta files. This could be due to the specific way the passive layer is modified, which manufacturers do not disclose.

In the current study, Fanta files immersed dynamically in NaOCl at 2.6% concentration lost significantly more Ni and Ti wt.% than new Fanta files or those immersed at 5.25% concentration. The reduction in Ni and Ti could be attributed to the corrosive effect of chloride ions in NaOCl solution. These findings agreed with the higher Ra and Rq values reported in Fanta files, as revealed by SEM-ImageJ analysis in this study. Azure files also exhibited the same pattern, but with no significant differences among different NaOCl exposures. The loss of Ni was greater than that of Ti, which is consistent with earlier research indicating that Ni is less stable than Ti and dissolves more easily [34].

In contrast, the C wt.% increased significantly in the Fanta files group after immersion in a 2.6% concentration. While most metal studies view the presence of C as a form of contamination, it has been suggested that elevated C concentrations may indicate the formation of titanium carbide. Research on the corrosion and passivity of NiTi shape memory alloys in biomedical applications found a correlation between reduced C levels and enhanced corrosion resistance. Carbon concentration affects the formation and distribution of titanium carbide particles. Additionally, the oxides at this interface are unstable and non-protective, rendering the metal susceptible to pitting [18]. Moreover, the oxygen content of Fanta files increased after exposure to NaOCl compared to Azure files, likely due to the lower accumulation of chloride ions on their surfaces. A reduction in chloride ions may promote the development of a more effective passivating film. Conversely, Azure files might have undergone localized chloride ion accumulation. The latter resulted in the disruption of the oxide layer and a subsequent decrease in O wt.% as shown by EDX analysis, unlike Fanta and the new Azure files. However, the detection of oxides in the EDX spectrum from corroded areas does not constitute definitive evidence of corrosion, as it may simply reflect passive film products [24].

To the best of our knowledge, no correlation has been established between increased roughness and clinical failure rates of used rotary files (Fanta and Azure blue heat-treated files) or comparable research. It was found that increased roughness in NiTi endodontic rotary files, particularly in the cutting blade, affects various mechanical parameters related to clinical performance during endodontic treatment. Increasing surface roughness elevates friction between the instrument and the tooth, thereby diminishing cutting efficiency, cyclic fatigue strength, and safety. Consequently, these surface imperfections may lead to stress concentration, crack propagation, and potential fracture during clinical use [27, 35].

However, the in vitro study's limitations included the use of a plastic container for immersion, which does not accurately replicate the surface interactions between the file and the canal walls. Variations in root canal morphology may impact mechanical stresses during instrumentation and influence the surface roughness of NiTi rotary instruments. Additionally, the dentin's buffering capacity against irrigants can alter the solution pH, as well as the room temperature, which differs from the root canal temperature. Hence, the clinical significance of these findings highlights the need for further research to assess clinical performance and investigate the corrosion fatigue failure of blue heat-treated NiTi files.

## Conclusion

Within the limitations of this in vitro study, Azure files demonstrated lower overall surface roughness than Fanta files after dynamic immersion in NaOCl at 2.6% NaOCl irrigant. This may warrant caution regarding the use of Fanta files; however, the in vitro nature of the study underscores the need for further investigation. Additional research is needed to establish a correlation between the study findings and fatigue failure.

## Data Availability

The datasets generated and analysed during the current study are available from the corresponding author on reasonable request.

## Abbreviations

NiTi: Nickel-Titanium
SEM: Scanning electron microscopy
EDX: Energy-Dispersive X-ray
Ra: Arithmetical mean roughness
Rq: Root mean square roughness
NaOCl: Sodium hypochlorite
SD: Standard deviation
Ni: Nickle
Ti: Titanium
Al: Aluminium
C: Carbon
O: Oxygen
N: Nitrogen
